# Exploiting cell death and tumor immunity in cancer therapy: challenges and future directions

**DOI:** 10.3389/fcell.2024.1416115

**Published:** 2024-06-03

**Authors:** Jiaan Lu, Ru He, Yang Liu, Jinghan Zhang, Heng Xu, Tianchi Zhang, Li Chen, Guanhu Yang, Jun Zhang, Jie Liu, Hao Chi

**Affiliations:** ^1^ Clinical Medical College, Southwest Medical University, Luzhou, China; ^2^ Department of Anesthesiology, Southwest Medical University, Luzhou, China; ^3^ Chengdu University of Traditional Chinese Medicine, Chengdu, China; ^4^ Department of General Surgery, Dazhou Central Hospital, Dazhou, China; ^5^ Department of Specialty Medicine, Ohio University, Athens, OH, United States

**Keywords:** non-apoptotic cell death, cellular signaling network, immunotherapy, combined therapy, immunogenic cell death, tumor microenvironment, TME, ROS

## Abstract

Cancer remains a significant global challenge, with escalating incidence rates and a substantial burden on healthcare systems worldwide. Herein, we present an in-depth exploration of the intricate interplay between cancer cell death pathways and tumor immunity within the tumor microenvironment (TME). We begin by elucidating the epidemiological landscape of cancer, highlighting its pervasive impact on premature mortality and the pronounced burden in regions such as Asia and Africa. Our analysis centers on the pivotal concept of immunogenic cell death (ICD), whereby cancer cells succumbing to specific stimuli undergo a transformation that elicits robust anti-tumor immune responses. We scrutinize the mechanisms underpinning ICD induction, emphasizing the release of damage-associated molecular patterns (DAMPs) and tumor-associated antigens (TAAs) as key triggers for dendritic cell (DC) activation and subsequent T cell priming. Moreover, we explore the contributions of non-apoptotic RCD pathways, including necroptosis, ferroptosis, and pyroptosis, to tumor immunity within the TME. Emerging evidence suggests that these alternative cell death modalities possess immunogenic properties and can synergize with conventional treatments to bolster anti-tumor immune responses. Furthermore, we discuss the therapeutic implications of targeting the TME for cancer treatment, highlighting strategies to harness immunogenic cell death and manipulate non-apoptotic cell death pathways for therapeutic benefit. By elucidating the intricate crosstalk between cancer cell death and immune modulation within the TME, this review aims to pave the way for the development of novel cancer therapies that exploit the interplay between cell death mechanisms and tumor immunity and overcome Challenges in the Development and implementation of Novel Therapies.

## 1 Background

Cancer is a major global health concern, responsible for millions of deaths annually. The International Agency for Research on Cancer (IARC) reports that cancer is the foremost or second-leading cause of death before the age of 70 in 112 out of 183 surveyed countries (Bray et al., 2021). Cancer cells possess unique characteristics enabling them to evade cell death and the immune system, making inducing cancer cell death an crucial aspect of therapy (Chi et al., 2022a; Zhao S. et al., 2022; Chi et al., 2022b).

The Nomenclature Committee on Cell Death (NCCD) has categorized cell death into accidental (ACD) and regulated (RCD) forms (Hanahan and Weinberg, 2011). RCD, orchestrated by specific molecular mechanisms, includes apoptotic and non-apoptotic variants such as ferroptosis, autophagy, pyroptosis, and necroptosis (Gao et al., 2022). Targeting non-apoptotic RCD pathways with drugs could overcome apoptosis resistance and impact cancer treatment (Muppa et al., 2019; Koren and Fuchs, 2021). Necroptosis, ferroptosis, and pyroptosis interact with tumor immunity in the TME, known as immunogenic cell death (ICD) (Tang R. et al., 2020; Shen et al., 2022; Song et al., 2022).

ICD in the TME activates anti-tumor immune responses, involving dendritic cells (DCs) presenting antigens to T cells, leading to cancer cell elimination and anti-tumor immunity development (Xie et al., 2017). Inducing necroptosis, ferroptosis, or pyroptosis in tumor cells can enhance anti-tumor immune responses and reduce tumor growth and metastasis (Xie et al., 2017; Tang R. et al., 2020; Chen et al., 2021a). This review explores the roles of both ICD (Immunogenic Cell Death) and non-apoptotic RCD (Regulated Cell Death) in the modulation of tumor immunity within the tumor microenvironment (TME). It proposes the TME as a viable target for innovative cancer therapies. Additionally, the aim is to provide readers with an understanding of the various forms of cell death induced by different therapies and their impact on anti-tumor immune responses. The review strives to examine the potential therapeutic approaches for enhancing and developing anti-tumor immunity through the utilization of these cell death mechanisms. Furthermore, it discusses the challenges associated with tumor resistance to cell death induction, the complexity of the tumor microenvironment, and the possible incorporation of new technologies to tackle these obstacles.

## 2 Immunogenic cell death, inflammation-associated pyroptosis, necroptosis, and ferroptosis

### 2.1 immunogenic cell death

**FIGURE 1:**
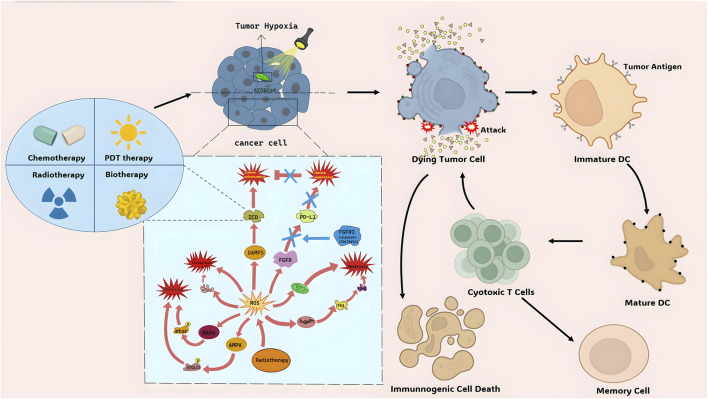
The mechanism of ROS and ICD.

ICD is a type of cancer cell demise triggered by certain treatments such as chemotherapeutic agents, oncolytic viruses, therapies, and radiotherapy (Ahmed and Tait, 2020). The non-immunogenic characteristics of tumor cells can transform into immunogenic traits upon exposure to these stimuli, leading to the production of anti-tumor immune responses (Galluzzi et al., 2020). ICD can be induced by intracellular pathogens, different types of drugs, and various physical therapies (Galluzzi et al., 2020). After ICD, cells release molecules known as DAMPs, TAAs, and pro-inflammatory cytokines. These molecules are captured by DCs and macrophages, processed, and presented to immune cells, ultimately resulting in antigen-specific immune responses (Duan et al., 2019). Pattern recognition receptors (PRRs) like Toll-like receptors (TLRs) and nucleotide-binding oligomerization domain-like receptors (NLRs) identify these molecules, stimulating tumor-specific immune responses. This process enhances the efficacy of anti-cancer medications by directly eliminating cancer cells and promoting anti-tumor immunity, including immune memory (Kroemer et al., 2022). Although pyroptosis, necroptosis, and ferroptosis can be considered forms of immunogenic cell death to some extent, they differ in their specific mechanisms and effects on immune system activation (Figure 1).

### 2.2 Inflammation-associated pyroptosis, necroptosis, and ferroptosis

Inflammation-associated pyroptosis is a form of cell death triggered by inflammasomes. It is characterized by cell swelling, rupture, and release of cellular contents (Hsu et al., 2021). Apoptotic caspases, including CASP1, CASP4, CASP5, CASP11, and murine CASP3, can also activate pyroptosis (Lee et al., 2023). NLRP3 inflammasomes are formed and caspase-1 is activated when pathogen-associated molecular patterns (PAMPs) and DAMPs bind to pattern recognition receptors. Caspase-1 cleaves gasdermin D (GSDMD), generating the N-terminal pore-forming domain (PFD) (Hou et al., 2020). The cleaved PFD of gasdermin induces oligomerization in the plasma membrane, resulting in cell death. It also promotes the maturation and release of active IL-1β and IL-18 (Holler et al., 2000; Tong et al., 2022). Lipopolysaccharide can directly activate CASP4, CASP5, and CASP11 to causes the above mechanism.

Necroptosis is another form of regulated cell death triggered by various factors, such as tumor necrosis factor (TNF), lipopolysaccharide (LPS), and nuclear DNA damage (Degterev et al., 2005; Galluzzi et al., 2017). Necroptosis shows morphological characteristics similar to necrosis, including organelle swelling, cell membrane rupture, and cytoplasmic and nuclear disintegration (Weinlich et al., 2017). Unlike apoptosis, necroptosis is caspase-independent. Under caspase-8 inhibition, receptor-interacting protein kinase 1 (RIPK1), RIPK3, and cylindromatosis (CYLD) remain active. The cellular death pathway can transition from apoptosis to necroptosis, leading to decreased cellular Adenosine triphosphate (ATP) levels (Temkin et al., 2006; Gong et al., 2019). Upon factors binding to cell membrane receptors, RIPK1 undergoes autophosphorylation and forms functional amyloid-like proteins, aided by Caspase-8 inhibition or E3 ligase family inhibitors. RIPK1 then interacts with RIPK3 to form the necrosome, initiating necroptosis. Phosphorylated mixed-lineage kinase domain-like (MLKL) translocates to the plasma membrane, forming an ion channel that increases membrane permeability and eventually leads to membrane rupture and DAMPs release, triggering necroptosis (Kaczmarek et al., 2013; Seo et al., 2021).

Ferroptosis is a non-apoptotic form of cell death characterized by the accumulation of lipid peroxidation (Dixon et al., 2012). It is dependent on iron and exhibits morphological changes, such as increased membrane density, mitochondrial shrinkage, reduced mitochondrial cristae, and normal nuclear morphology without chromatin condensation (Stockwell et al., 2017). Ferroptosis can be induced by various factors. Glutathione peroxidase 4 (GPX4) inhibits the generation of active lipid oxygen, but when the cystine-glutamate transporter (system xc-) is inhibited, the uptake of glutathione (GSH) decreases, leading to decreased GPX4 activity and initiation of ferroptosis (Yang et al., 2014). p53 can also trigger ferroptosis by downregulating the expression of SLC3A2, decreasing GSH synthesis and GPX4 activity (Chu et al., 2019). RSL3, DPI10, and mevalonate pathway-targeting molecules directly influence GPX4 and induce ferroptosis (Yu et al., 2017; Chen et al., 2021b). When iron is present, the peroxidation of polyunsaturated fatty acids (PUFAs) in cellular membrane lipids generates reactive oxygen species that oxidize adjacent PUFAs, resulting in membrane phospholipid peroxidation and ferroptosis (Lee et al., 2021). Components of the autophagy machinery, such as ATG3 and ATG5, can also activate ferroptosis (Zhou et al., 2020). Although the immunogenicity of ferroptosis is not extensively studied, preliminary evidence suggests that it may trigger immune responses by releasing DAMPs (Wen et al., 2019; Tang D. et al., 2020).

## 3 Immunity and tumor cell death

Immune checkpoint inhibitors (ICIs) have revolutionized cancer therapy, but many tumors do not respond well, it is often due to low levels of tumor-infiltrating lymphocytes (TIL). This limitation hinders the broader use of ICIs in immunotherapy (Chi et al., 2022b). TILs reside in the TME, which surrounds tumor cells and significantly impacts tumor progression through various secreted factors. The TME includes stromal cells, immune cell populations (T and B lymphocytes, neutrophils, TAMs et al.), among others (Junttila and de Sauvage, 2013). Modulating the TME and TIL composition by inducing immunogenic cell death (e.g., necroptosis, ferroptosis, or pyroptosis) shows promise in enhancing anti-tumor immunity (Niu et al., 2022).

### 3.1 Immunogenic cell death

During immunogenic cell death in cancer cells, various substances enhance the immune response and kill more tumor cells (Xie et al., 2022). High mobility group box 1 protein (HMGB1), alone or in combination with Toll-like receptors and receptor for advanced glycosylation end-products (RAGE), promotes inflammatory reactions (Although it mainly exists in the serum of arthritis patients and at the inflammatory sites of patients with septicemia, some studies suggest that it may also be a therapeutic target for cancer patients.), produces pro-inflammatory cytokines, and enhances antigen presentation by DCs, resulting in powerful anti-tumor immune effects (Lotze and DeMarco, 2003; van Beijnum et al., 2008). Calreticulin (CRT) engagement with CD91 fosters the maturation and activation of DCs, culminating in the cross-presentation of tumor antigens and the elicitation of tumor-specific cytotoxic T lymphocyte (CTL) responses. This progression concurrently stimulates the secretion of pro-inflammatory cytokines such as TNF-α and IL-6, bolstering the anti-tumor immune response through diverse mechanisms (but total or membrane-exposed CALR levels are closely associated with prognosis in different cancer types, such as patients with myeloproliferative neoplasms carrying CALR mutations showing better outcomes compared to patients with wild-type CALR.) (Pawaria and Binder, 2011; Fucikova et al., 2021). The binding of ATP to its receptor also triggers the activation of cytotoxic T lymphocytes (CTLs), propels DC activation and maturation, and expands macrophage populations (Elliott et al., 2009; Troitskaya et al., 2022).

### 3.2 Inflammation-related pyroptosis

In the immune defense mechanism, pyroptosis occurs more frequently than necroptosis and ferroptosis (Hsu et al., 2021). Gasdermin (GSDM) proteins act as key executors of pyroptosis, directly inducing cancer cell lysis and the release of immune-stimulating cellular contents. Additionally, they aggregate and activate immune cells such as CD4 andCD8 T cells, thus promoting tumor cell death (CT26 colorectal carcinoma and B16 melanoma in mouse) (Kong and Zhang, 2023). Pyroptosis transforms the immunosuppressive “cold” TME into an immunogenic “hot” TME, facilitating the infiltration of tumor-infiltrating lymphocytes (Zhu and Li, 2023). A recent study demonstrated that delivering metal ions and immune adjuvant R848 together to the tumor tissue can enhance anti-tumor immunity through pyroptosis regulation (Feng et al., 2023). NLRP3 inflammasome activation can also promote intestinal epithelial cells to secrete IL-18, exerting an anti-tumor effect on immune cells such as CD4^+^ T cells (in AOM-induced colorectal cancer) (Du et al., 2016).

### 3.3 Necroptosis

Currently, there are two ways to induce anti-tumor immunity through necroptosis: using necroptotic tumor cells or fibroblast vaccines (Tang R. et al., 2020). Aes et al. (2016) demonstrated that necroptotic cells release DAMPs, which activate DCs, promote antigen presentation, and stimulate cytotoxic CD8^+^ T lymphocytes (Aaes et al., 2016). However, recent research has highlighted the crucial roles of BATF3+ cDC1 cells and CD8^+^ leukocytes in tumor control mediated by necroptotic cells (Snyder et al., 2019). Transplanting necrotic cells into the TME can activate BATF3+ cDC1 cells and CD8^+^ leukocytes via necroptosis, leading to a powerful immune response independent of DAMPs released by MLKL. In this process, NF-κB activation in dying cells is essential for initiating immune responses and facilitating interactions between necroptosis and the TME (.F10-OVA melanoma flank tumors and Lewis Lung (LL/2)-OVA adenocarcinoma flank tumors) (Snyder et al., 2019). Furthermore, RIPK3 activation in cancer cells can also induce TRIM3 to modulate and enhance the anti-tumor microenvironment (Park et al., 2021).

## 4 TME as a therapeutic target for cancer treatment

### 4.1 Immuno-stimulated cell death

#### 4.1.1 Combined therapy targeting immuno-stimulated cell death

The TME is a potential target for cancer treatment by inducing ICD in tumor cells. Immune Checkpoint Blockade (ICB) therapy activates the immune system to target cancer cells by inhibiting immune checkpoint molecules on tumor cell surfaces (Xie et al., 2022). However, the Immune-Suppressive Tumor Microenvironment (ITM) can limit the effectiveness of ICB therapy. Combining ICB therapy with chemotherapy or Photothermal Therapy (PTT) and Photodynamic Therapy (PDT) enhances intratumoral cytotoxic T lymphocyte (CTL) infiltration and overcomes ITM constraints. ICG-mediated PTT facilitates the uniform distribution of nanoparticles (NPs) in tumor tissues, enhancing drug efficacy (Li et al., 2017). PDT directly damages tumor DNA and induces tumor cell autophagy, showing synergistic effects with targeted drugs (Robertson et al., 2009; Zhang et al., 2016; Wen et al., 2017; Wang et al., 2018). Additionally, PDT-assisted treatments reduce tumor cell oxygen consumption and block electron transfer chains (Zhao et al., 2020) (Figure 1). Chemotherapy or PDT induce tumor cell apoptosis, release antigens, and activate the immune system, promoting CTL activity. This combined therapy improves treatment efficacy and reduces side effects. Furthermore, nanodrug delivery systems targeting ITM, like NPs, enhance the effectiveness of ICB therapy. Loading immune checkpoint inhibitors into nanocarriers improves their bioavailability in tumor tissues, overcoming limitations of the multiple types of tumor microenvironment. These strategies offer promising avenues for cancer treatment.

#### 4.1.2 The sword of radiation therapy–reactive oxygen species (ROS) system

Radiation therapy (RT) uses high-energy radiation to damage cancer cells and inhibit their growth. It can generate ROS to break down cellular components and cause tumor cell death (Ozben, 2007) (Figure 1). ROS produced by RT can trigger tumor cell death through different pathways.

1) Inducing tumor cell apoptosis: ROS can activate the P53 pathway, increase pro-apoptotic proteins, and induce cell apoptosis. It can also affect mitochondrial integrity, leading to apoptotic cell death. ROS can also activate death receptor ligand pathways to trigger cell apoptosis (human leukemia HL-60 cells) (Ho et al., 2011).
2) Inducing tumor cell autophagy: ROS can activate the AMPK and MAPK pathways, promoting the expression of autophagy-related proteins and autophagosome formation, leading to autophagic cell death (human gastric cancer) (Liu and Fan, 2019).
3) Inducing tumor cell ferroptosis: ROS can enhance lipid peroxidation, particularly of polyunsaturated fatty acids, leading to tumor cell death via ferroptosis. ACSL4 is an important regulator in this process (Multiple types of cancer.) (Yang et al., 2022).
4) Augmenting ICD: ROS induced by RT can stimulate the release of DAMPs, activating immune cells and enhancing tumor recognition and clearance by the immune system. ROS can also modulate the function and activation of immune cells within the tumor microenvironment (triple-negative breast cancer *in vitro* and *in vivo*) (Krombach et al., 2019).

ROS can also affect immune checkpoint molecule expression, such as PD-L1, in immune cells (Glorieux et al., 2021; He et al., 2024). Additionally, ROS can regulate T cell function and apoptosis, impacting immune checkpoint expression and immune cell activity (colorectal cancer) (Kesarwani et al., 2013).

In summary, ROS induced by RT can promote tumor cell death through multiple mechanisms and modulate immune cell function. They can also alter the tumor microenvironment and induce immunogenic tumor cell death.

#### 4.1.3 Peptides immunoactivation of the tumor microenvironment

Therapeutic peptides are potent agents for eliminating bacteria and tumor cells, impacting immune system activity. They play a significant role in immune modulation by circumventing limitations of conventional treatments. LTX-315 is a novel therapeutic peptide known for inducing ICD. Zhou et al. (2016) used several types of fibrosarcomas on mice and they found It induces structural and functional changes in mitochondria, leading to cell death and the release of DAMPs. ACPP, another pro-apoptotic peptide, enhances macrophage phagocytic activity and promotes anti-tumor immune responses by disrupting the CD47-SIRPα interaction according to a study in mouse colon cancer cells (Koh et al., 2017; Wang et al., 2021). Peptides targeting various cells and signaling pathways within the TME are being investigated to boost immune activity against tumors (Table 1). Continued research on these peptides offers promising avenues for cancer treatment (Hayes et al., 2020; Aria and Rezaei, 2023).

**TABLE 1:** Other ICD-inducing peptides.

Peptide	Mechanism of action	Effects in cancer immunotherapy	References
RL2	Inhibits TOM70 on mitochondrial membrane, reducing ATP production and inducing necrosis	- Effective against various tumors	Troitskaya et al. (2020)
		- Triggers immunogenic cell death (ICD) in cancer cells	
		- Increases phagocytosis and anti-cancer immunity	
F-pY-T	Targets mitochondria, generates oxidative stress, triggers ICD, increases ATP secretion	- Enhances anti-cancer immunity when combined with immune checkpoint inhibitors (ICIs)	(Zheng et al., 2022) (The experiment used CT26 tumors implanted in mice.)
		- Boosts CD8^+^ T cell penetration into the tumor microenvironment (TME)	
CBP501	Inhibits calmodulin, sensitizes cancer cells to cisplatin-induced ICD	- Enhances efficacy of platinum-based therapy	(Mine et al., 2011) (non-small cell lung cancer and malignant pleural mesothelioma)
		- Increases CD8^+^ T cell penetration into TME	
		- Modulates tumor-associated macrophage (TAM) populations, improving anti-tumor responses	
RT53	Binds to API5 on tumor cell membranes, inhibits pro-survival activities, induces necrosis	- Induces ICD hallmarks such as HMGB1 and ATP release	(Pasquereau-Kotula et al., 2018; Habault et al., 2020) (B16F10 melanoma cellsandacute promyelocytic leukemia (APL) in mouse)
		- Increases T cell infiltration and anti-tumor responses	
PKHB1	Binds to CD47, induces calcium-dependent and caspase-independent cancer cell death	- Induces ICD markers	(Uscanga-Palomeque et al., 2019; Calvillo-Rodríguez et al., 2022) (CEM and MOLT-4 human cell lines (T cell acute lymphoblastic leukemia; T-ALL) and on one T-murine tumor lymphoblast cell-line (L5178Y-R) (T-ALL))
		- Enhances leukocyte infiltration at tumor sites	
		- Stimulates immunological memory, suppressing tumor growth	

### 4.2 Role of inflammation-associated cell pyroptosis and necroptosis in enhancing tumor immunogenicity

Pyroptosis and necroptosis are programmed cell death mechanisms that hold potential in cancer therapy by enhancing tumor immunogenicity and immune cell infiltration in the TME. Pyroptosis, triggered by gastrin-releasing peptide, can transform “cold tumors” into “hot tumors” by augmenting the immune response (Zhang R. N. et al., 2023). Colorectal cancer (CRC) treated with gambogic acid (GA) amplifies the ratio of immune cells such as CD3 T cells, cytotoxic T lymphocytes, dendritic cells, and effector memory T cells, promoting Pyroptosis and enabling chemotherapy drugs to modulate the TME and improve antitumor efficacy (Xu et al., 2022).

Various drugs, including metformin, anthocyanins, and dehydroacetic acid, are capable of inducing Pyroptosis and potentiating anticancer immunity (on human hepatocellular carcinoma,naïve breast cancer and et al.) (Paixão et al., 2017; Kheirandish et al., 2018; Zhou et al., 2018). However, cancer cells can evade Pyroptosis through immune suppression pathways and drug resistance mechanisms. To address this issue, Feng et al. developed an acid-responsive Fe/Mn bimetallic organic framework nanosystem loaded with metal ions and an immune adjuvant R848 (FeMn@R@H). This nanosystem initiates ROS-mediated Pyroptosis via Fenton-like reaction, reverses the suppressive tumor immune microenvironment, and amplifies antitumor immune therapy (Feng et al., 2023). Organic photosensitizers (PS) materials have also shown potential in provoking Pyroptosis and reshaping the TME (Zhou et al., 2018). Oroxylin A (Ori) is another compound that induces Pyroptosis and modulates the TME, demonstrating broad-spectrum anticancer effects (Sun X. et al., 2023).

Insufficient immune cell infiltration in the various TMEs limit the effectiveness of tumor immunotherapy. Radiotherapy, chemotherapy, and targeted therapy can enhance tumor cell immunogenicity, leading to immune cell infiltration and tumor cell demise (Galon and Bruni, 2019; Kroeze et al., 2023; Park et al., 2023). Chemotherapy and radiotherapy-induced acute necroptosis enable DCs to present antigens and activate T cells, resulting in cytotoxic T cell infiltration in the activated TME and tumor eradication. Fucoidan and 5-FU can bolster anticancer immunity by provoking necroptosis and has been widely used and effective in various solid tumors (Zhao et al., 2017; Wang H. et al., 2019). Combination therapy utilizing chemotherapy and immune checkpoint inhibitors (ICI) has shown promising results in cancer treatment and the effects were significantly better than the control group in a randomized, open-label three-phase experiment (Reck et al., 2019). In addition, Polo-like kinase 1 (PLK1) has been identified as a potential inhibitor of tumor immunity and necroptosis. Inhibiting PLK1 may serve as a promising therapeutic strategy (Zhang P. et al., 2023).

In summary, both Pyroptosis and necroptosis play important roles in enhancing tumor immunogenicity and promoting immune cell infiltration in the TME. Strategies involving the induction of Pyroptosis and necroptosis, combinational therapies, and targeting PLK1 hold promise in cancer therapy. It can also trigger deeper thinking for us. Lipid peroxidation induced by oxygen free radicals plays a negative role in the theory of cellular aging. However, in various tumor microenvironments, the induced lipid peroxidation can induce tumor cell programmed death through iron death pathways. This suggests that we can only better utilize the mechanisms of cellular metabolism and survival to benefit the health of all mankind by correctly understanding and further researching them.

### 4.3 Potential strategies for utilizing ferroptosis as a therapeutic target

Ferroptosis promotes immune cell recruitment and enhances anti-tumor immune response (Chu et al., 2019; Wen et al., 2019). CD8^+^ T cell activation by immune therapy downregulates SLC7A11 expression, inducing cancer cell ferroptosis in patients with melanoma (Wang W. et al., 2019). NRF2 nanomodulators induce ferroptosis in lung cancer cells and stimulate the TME to initiate immune response (Hsieh et al., 2021). Inhibiting MKL-1 expression enhances sensitivity to ferroptosis-inducing agents in TME of gastric cancer (Dai et al., 2023). Chemotherapy drugs and radiotherapy also induce ferroptosis, improving the efficacy of immune checkpoint inhibitor (ICI) immunotherapy by promoting immune cell infiltration and increasing tumor cell immunogenicity (Tong et al., 2022).

Ferroptosis, mediated by lipid peroxidation, aids in tumor antigen recognition and processing by DCs, promoting CD8^+^ T cell presentation and cytotoxic lymphocyte activation, enhancing tumor immunotherapy (Zhao L. et al., 2022). Combining immune checkpoint inhibitors (ICIs) with ferroptosis inducers may increase various tumor cells sensitivity to immune therapy. Several FDA-approved drugs, including glutamine, sorafenib, cisplatin, gemcitabine, and linsitinib, induce ferroptosis and are potential candidates for cancer treatment (Dixon et al., 2014; Stockwell et al., 2017; Guo et al., 2018; Lee et al., 2020; Lv et al., 2020). However, drug resistance is a challenge. Multifunctional ferroptosis nanomedicines have shown promise in effectively reversing treatment resistance (Cai et al., 2024). For example, miR-654-5p enhances sorafenib-induced ferroptosis by targeting HSPB1, improving therapeutic outcomes in Sora-resistant hepatocellular carcinoma (HCC) patients when combined with m654-sEV (Sun J. et al., 2023). Carrier-free nanocomponents containing sorafenib (Sor) and gambogic acid (GA) deplete GSH, inducing ferroptosis *in vitro* and *in vivo*, exhibiting potent antitumor activity (Lei et al., 2023). A photoactivated oxygen self-supplying chemical photothermal nanoplatform enhances ferroptosis and alleviates hypoxia-induced chemoresistance in colorectal cancer (CRC) (Jiang et al., 2023). Phenanthroindolizidine alkaloids, natural products that inhibit various cancers, show promise in overcoming tumor resistance (Peng et al., 2023). Combining carbon ion radiotherapy (CIRT) with immunotherapy enhances immune cell infiltration, suggesting a potential role for ferroptosis in synergistic anticancer effects during CIRT combined therapy (Huang Q. et al., 2023).

## 5 Discussion

### 5.1 The complexity of cell death

Cell death, particularly ICD and its related forms like inflammation-associated cell pyroptosis, necroptosis, and ferroptosis, plays a key role in cancer development and treatment. These types of cell death stimulate the immune system and trigger crucial anti-tumor responses.

ICD can convert non-immunogenic tumor cells into immunogenic ones, prompting the body’s anti-tumor immune reaction. Released molecules like DAMPs, tumor-associated antigens (TAA), and pro-inflammatory cytokines are captured by DCs and macrophages, initiating antigen-specific immune responses that bolster anti-tumor immunity. Research has found that the inhibition of autophagy can also trigger the accumulation of DAMPs, thereby inducing ICD (Michaud et al., 2011). Clinical use of autophagy inhibition mechanism has shown effective treatment against tumors (Di Lernia et al., 2020). However, further research is needed on the specific pathways and regulatory factors through which autophagy modulation mediates immunogenic cell death. Exploring novel autophagy inhibitors and combination strategies holds promise for improving therapeutic outcomes and represents one of the challenges in the next Frontier of cancer treatment. Similarly, inflammation-associated cell pyroptosis, necroptosis, and ferroptosis exhibit immunogenic properties and can activate the immune system by releasing immune-stimulating substances. And ultimately fostering anti-tumor immune responses (Tang R. et al., 2020).

Yet, the intricate interplay between cell death and the TME cannot be overlooked. The TME is a complex ecosystem comprising tumor cells, immune cells, stromal cells, blood vessels, and others. Cell death not only alters the TME composition but also regulates it. Immune responses induced by ICD can modify the immune landscape of the TME, restraining tumor growth and metastasis. However, considering the TME’s complexity, understanding the molecular mechanisms governing the interaction between cell death and the TME is crucial to optimizing treatment strategies (Kesarwani et al., 2013; Zhou et al., 2016).

### 5.2 Future direction

Future research should delve deeper into the molecular mechanisms of various forms of cell death, especially key molecules like CRT (Pawaria and Binder, 2011), HMGB1 (van Beijnum et al., 2008), and ATP (Troitskaya et al., 2022) involved in the ICD process. Quantitative and spatial analysis of cell death are also pivotal in refining treatment strategies such as tailoring and monitoring personalized immunotherapy regimens (Banna et al., 2019). Additionally, leveraging advanced technologies and tools like gene editing and nanotechnology to modulate cell death processes presents promising avenues for enhancing cancer treatment efficacy (Li et al., 2017; Dilnawaz and Acharya, 2023).

In essence, the role of cell death in cancer treatment is multifaceted, encompassing the tumor microenvironment, immune responses, and treatment strategies. Future research should focus on delving into these facets and embracing novel technologies and methodologies to tackle the challenges in cancer treatment, ultimately elevating treatment outcomes and patients’ quality of life.

### 5.3 The detection methods for treating benefit-based patients (such as single-cell analysis)

Through the discussion of various therapeutic approaches inducing programmed cell death and immune cell death, we have discovered that many treatments have strong limitations, benefiting only certain tumor types with specific characteristics, such as patients with myeloproliferative neoplasms carrying CALR mutations showing better outcomes (Fucikova et al., 2021; Huang X. et al., 2023; Chi et al., 2023; Zhao et al., 2023). Therefore, it is crucial to effectively utilize current hot topics like single-cell analysis and other diagnostic tools to classify patients who can or cannot benefit from treatment earlier, faster, and more accurately through cell markers. Many detection models based on single-cell and other technologies have been developed to quickly detect and automatically diagnose various cell types, including but not limited to acute leukemia, breast cancer cell biomarkers, and mature T cells (Zhou et al., 2015; Tsai et al., 2020; Wang et al., 2022).
